# The impact of the COVID-19 pandemic on forensic pathology services in Limpopo province, South Africa

**DOI:** 10.4102/safp.v67i1.6038

**Published:** 2025-01-29

**Authors:** Thakadu A. Mamashela, Samuel T. Ntuli

**Affiliations:** 1 Department of Forensic Pathology, School of Medicine, University of Limpopo, Limpopo, South Africa; 2 Department of Statistical Sciences, Faculty of Science and Technology, Sefako Makgatho Health Sciences, Pretoria, South Africa

**Keywords:** COVID-19, pandemic, forensic, autopsy, Limpopo province, South Africa

## Abstract

**Background:**

To evaluate the effects of coronavirus disease 2019 (COVID-19) and the preventative measures taken, especially how they affect forensic pathology services in rural South Africa.

**Methods:**

This retrospective analysis includes referred post-mortem cases from all forensic pathology services in Limpopo province, comparing the period before the COVID-19 pandemic (01 January 2019 to 31 December 2019) with the pandemic period (01 January 2020 to 31 December 2020). Data analysis was performed using STATA 16.0 software (StataCorp; College Station, TX). Chi-square test was employed for comparison, with a *p*-value < 0.05 deemed statistically significant.

**Results:**

Approximately 9319 cases were submitted for post-mortem examinations, with 4857 occurring before the pandemic and 4462 during it, marking an 8.1% decrease. There was a decrease in the number of unnatural death cases, while the instances of natural deaths rose. Cases under investigation saw a notable increase. There was a marked decrease in referrals for forensic examinations across all districts. In addition, except for one facility, there was a decline in the number of cases sent for autopsies at all facilities.

**Conclusion:**

In conclusion, forensic pathology services in this province had been severely disrupted by the COVID-19 outbreak and the lockdown that followed, especially in the tertiary hospital. It has led to new challenges for case management and necessitated changes to operating procedures.

**Contribution:**

It has required modifications to operational procedures and has introduced various challenges in case management.

## Introduction

Coronavirus disease 2019 (COVID-19) was first identified in Wuhan City, Hubei province, China, on 12 December 2019,^1^ and was declared a global pandemic by the World Health Organization on 11 March 2020.^2^ In response to the escalating pandemic, many countries, including South Africa, implemented public health interventions such as social distancing, mask-wearing, self-isolation, closure of businesses and schools, travel restrictions, bans on public events, and regulated movement of people.^3^ These measures have led to a variety of outcomes in South Africa, notably a reduction in non-natural deaths, but an increase in mortality because of natural causes.^4^ A similar trend has been observed in research conducted by Gunawardena et al. in Colombo, Sri Lanka.^5^

Forensic pathology services are pivotal in unravelling the details surrounding deaths, pinpointing causes, and determining the timing of these events.^6,7,8^ They have been instrumental in advancing our understanding and surveillance of diseases, aiding in both prevention and treatment strategies.^9,10^ Despite the World Health Organization and the Centers for Disease Control and Prevention (CDC) setting forth clear guidelines for handling deceased individuals during the COVID-19 pandemic,^11,12^ there remained apprehensions. Varied responses were observed globally, with some nations foregoing clinical autopsies and others conducting them with reduced comprehensiveness.^13,14,15^

During the pandemic, low- and middle-income countries, including South Africa (SA) encountered considerable challenges, such as elevated infection rates and a scarcity of resources. Like other developing countries, SA experienced a lack of adequate personal protective equipment,^16^ which has led to fewer comprehensive autopsies being conducted.^13^ Despite the challenges faced, there is still a significant gap in empirical research evaluating the effects of the COVID-19 pandemic on forensic pathology services in the Limpopo province. Therefore, the purpose of the present investigation was to investigate how COVID-19 pandemic has affected forensic pathology services in the South African province of Limpopo.

## Methods

### Study design and setting

The study entails a retrospective analysis of referred post-mortem cases across all forensic pathology mortuaries in Limpopo province. It spans from 01 January 2019 to 31 December 2019 (pre-COVID-19 pandemic) and from 01 January 2020 to 31 December 2020 (during the pandemic). The province hosts 12 forensic pathology facilities distributed within hospitals across its five districts. Among these, one is stand-alone, six are in district hospitals, four in regional hospitals, and one in a tertiary hospital. Each district, except for district B, possesses two such facilities.

### Study population

The study encompassed all forensic post-mortem cases referred to and examined during the COVID-19 lockdown, as well as those during the corresponding period in the non-pandemic year.

### Data collection

Data for this study were collected by trained data collectors, specifically forensic pathology officers, who extracted information from the death register and recorded it directly into a Microsoft Excel Spreadsheet. The data collection process was supervised by the first author, a forensic pathologist, who performed quality checks and addressed any errors or inconsistencies. In addition, the forensic pathologist verified the assignment of the external cause of injury in certain instances.

### Data analysis

The statistical analysis was conducted with STATA 16.0 software (StataCorp; College Station, TX, United States), presenting results as counts and percentages. The Pearson Chi-square test was employed for variable comparisons. A *p*-value < 0.05 was deemed to indicate statistical significance.

### Ethical considerations

The research initiative received ethical approval from the University of Limpopo (reference number: TREC/326/2020: PG) and given permission to proceed by the Limpopo Provincial Department of Health (reference number: LP_2024-01-002).

## Results

A total of 9319 cases referred for post-mortem examinations, all of which were incorporated into the analysis. Among these cases, 4857 were reported before the pandemic, while 4462 were reported during the pandemic, marking an 8.1% decrease. Table 1 shows a significant reduction of 10.7% in unnatural deaths, decreasing from 4106 pre-COVID-19 to 3666 during the pandemic (*p* < 0.05). Likewise, undetermined deaths fell by 14.2%, from 190 prior to the pandemic to 163 during it, although this result was not statistically significant (*p* > 0.05). Conversely, there was a slight increase in the rate of natural deaths during the pandemic (9.1% compared to 8.1% pre-pandemic, *p* > 0.05), and the cases pending investigation rose from 3.4% to 5.0% (*p* < 0.05). An analysis of forensic examination referrals by district revealed a downward trend across all districts. Despite this overall decline, the decrease did not reach statistical significance in any district, as indicated by *p*-values > 0.05.

**TABLE 1:** Cases referred to forensic pathology mortuaries during the study period.

Variables	2019 (*n* = 4857)		2020 (*n* = 4462)		% Change (2019 vs. 2020)	*p*
	*n*	%	*n*	%		
Natural deaths	395	8.1	408	9.1	3.3	0.082
Unnatural deaths	4106	84.5	3666	82.2	−10.7	0.002
Undetermined	190	3.9	163	3.7	−14.2	0.513
Under investigations	166	3.5	225	5.0	35.5	< 0.001
**District**						
A	1848	38.0	1774	39.7	−4.0	0.091
B	834	17.2	721	16.2	−13.5	0.190
C	801	16.5	716	16.0	−10.6	0.561
D	542	11.2	480	10.8	−11.4	0.535
E	832	17.1	771	17.3	−7.3	0.849

Table 2 presents the outcomes of a subgroup analysis within institutions, showing that District A observed a significant increase in the number of cases forwarded for investigation at forensic pathology service at a tertiary hospital (Fps A1), as indicated by a *p* < 0.05. In contrast, forensic pathology service at a district hospital (Fps A2) saw a significant decrease in case referrals of 19.6%, also with a *p*-value < 0.05. The remaining districts experienced notable decreases in the number of cases sent for investigation; however, these were not statistically significant, evidenced by *p*-values exceeding 0.05.

**TABLE 2:** Cases referred for forensic pathology investigation according to the type of facility.

District	Facility	2019		2020		% change	*p*
		*n*	%	*n*	%		
A	FpsA1	1278	26.3	1316	29.5	3.0	< 0.001
	FpsA2	570	11.7	458	10.3	−19.6	0.024
B	FpsB1	405	8.3	367	8.2	−9.4	0.851
	FpsA2	157	3.2	119	2.7	−24.2	0.112
	FpsB3	151	3.1	138	3.1	−8.6	0.169
	FpsB4	121	2.5	97	2.2	−19.8	0.337
C	FpsC1	480	9.9	429	9.6	−10.6	0.675
	FpsC2	321	6.6	287	6.4	−10.6	0.737
D	FpsD1	248	5.1	240	5.4	−3.2	0.577
	FpsD2	294	6.1	240	5.4	−18.4	0.167
E	FpsE1	571	11.8	552	12.4	−3.3	0.373
	FpsE2	261	5.4	219	4.9	−16.1	0.325

Fps, forensic pathology services.

## Discussion

The study focused on assessing the effects of the COVID-19 pandemic on forensic pathology services within a rural province in SA. The findings of this study contrast with previous studies that observed an increase in the number of cases requiring post-mortem examination,^5^ yet they concur with results from other studies,^17,18,19^ indicating a complex and multifaceted influence of the pandemic on forensic services. Our research corroborated the expected increase in natural deaths during the pandemic, as well as a notable decline in unnatural deaths. In contrast, some studies have observed a reduction in both natural and unnatural deaths amid the restrictions.^18^ This might be because of the study being carried out at a single facility with comparable data from before and during the lockdown. Consistent with our results, several studies have documented a significant decrease in unnatural deaths during the pandemic.^4,5,17,20^ The rise in natural deaths observed in our study may be associated with hospital reports of COVID-19 fatalities requiring autopsies because of claims of medical misconduct during the pandemic. In addition, the observed decrease in unnatural deaths could be because of a decline in road traffic accidents and homicides, possibly because of the ban on sales and consumption of alcohol.^21^

It should be noticed that the post-mortem cases under scrutiny increased remarkably during the period of COVID-19 pandemic in our study. This may result from an unforeseen death that occurred at home, either with a prior medical history or acute symptoms, and the post-mortem examination fails to disclose any apparent reasons of death. Consequently, additional tests, including microscopic inspection of tissue samples from organs and toxicological analysis of blood and other fluids, were necessary to exclude poisoning. Furthermore, inadequate proficiency in conducting autopsy on fatalities caused by infectious diseases was reported, particularly those transmitted asymptomatically.^22,23^ As a result, an enhanced investment in pathology and forensic training and practice is essential. Our research indicates a decline in the number of cases referred to post-mortem examinations across various facilities, with the sole exception of one facility located in tertiary hospital. This observation contrasts with a report from a tertiary hospital in India, which found a decrease in post-mortem cases following the pandemic.^19^ The increase in autopsies at this single facility could be because of its association with a tertiary care centre, which typically has forensic expertise, post-mortem referral of cases or it might stem from a lack of expertise at other facilities in performing autopsies on deaths caused by infectious diseases, particularly those that are transmitted without symptoms. Moreover, the high level of expertise at tertiary care centres may account for the specialised services they provide. Such a facility is often part of a larger healthcare system, offering a higher level of care that often involves intricate medical procedures and treatments that require specialised abilities and equipment.

## Conclusion

In conclusion, forensic pathology services throughout all districts of this remote province have declined during the COVID-19 epidemic, affecting nearly all facilities. The facility in the tertiary hospital (FpsA1) was under great strain. This has resulted in new challenges for case management and required modifications to operating procedures. Notable increases in natural deaths may have contributed to a rise in medico-legal disputes, particularly in cases of hospital deaths linked to COVID-19. This circumstance emphasises how urgently specialised assistance is needed to handle the medico-legal problems resulting from allegations of medical malpractice during the pandemic.
